# Supplementary material for: A single point mutation in the Plasmodium falciparum FtsH1 metalloprotease confers actinonin resistance
Source: eLife. 2020 Jul 17;9:e58629. doi: 10.7554/eLife.58629 (PMC7386903; doi:10.7554/eLife.58629)
Supplement: Supplementary file 1. — (a) Screen of D10 clones for Pfftsh1 sequence and actinonin resistance. (b) Descriptive statistics for growth inhibition trials in Figure 1. (c) Oligos for PCR amplification of potential actinonin targets. (d) Oligos for generation and sequencing of allelic replacement constructs. [file elife-58629-supp1.docx]

**Supplementary File 1**

| Supplementary File 1a. Screen of D10 clones for *Pfftsh1* sequence and actinonin resistance. | | |
| --- | --- | --- |
| Act^R^ clone | **Nucleotide sequence of PF3D7_1239700 (*Pf*FtsH1)** | **Actinonin IC_50_**  **(µM)** |
| *Pf* D10 WT | 1451 CAGAAACTAGTAGT**G**GTGCTTCAAGTGATA 1480 | 2.0 ± 0.2 (n=4) |
| *Pf* D10 Act^R^ Clone 1 | 1451 CAGAAACTAGTAGT**T**GTGCTTCAAGTGATA 1480 | 73.3 ± 2.7 (n=3) |
| *Pf* D10 ACT^R^ Clone2 | 1451 CAGAAACTAGTAGT**T**GTGCTTCAAGTGATA 1480 | 61.6 ± 14.4 (n=2) |
| *Pf* D10 ACT^R^ Clone3 | 1451 CAGAAACTAGTAGT**T**GTGCTTCAAGTGATA 1480 | 51.4 (n=1) |
| *Pf* D10 ACT^R^ Clone4 | 1451 CAGAAACTAGTAGT**T**GTGCTTCAAGTGATA 1480 | 36.2 (n=1) |

**Supplementary File 1b: Descriptive statistics for growth inhibition trials in Figure 1.**

|  | D10 Act^S^ | D10 r*ftsH1***^G^**^489^**^G^** | D10 r*ftsH1***^G^**^489^**^C^**a | D10 r*ftsH1***^G^**^489^**^C^**b | D10 Act^R^ |
| --- | --- | --- | --- | --- | --- |
| n | 4 | 6 | 3 | 5 | 3 |
| Mean | 2.024 | 2.894 | 43.88 | 59.19 | 73.29 |
| Median | 2.117 | 2.887 | 43.60 | 58.71 | 71.69 |
|  |  |  |  |  |  |
| SD | 0.32 | 0.68 | 3.61 | 3.78 | 4.64 |
| SEM | 0.16 | 0.28 | 2.08 | 1.70 | 2.68 |
| 95% CI | 1.52-2.53 | 2.18-3.61 | 34.92-52.84 | 54.49-63.88 | 61.76-84.82 |

**Supplementary File 1c:** **Oligos for PCR amplification of potential actinonin targets.**

| GeneID | Forward primer | Reverse primer |
| --- | --- | --- |
| PF3D7_0907900  *Pfpdf* | TCACTCAGGAAAACCACAACT | CCCCCAGAAACAAAAGAACA |
| PF3D7_1313200  *Pfftsh1* | TCCAATCTAAGAAATATTCGACCCCT | AATGGAGAGAATTCTATGCCTCTT |
| PF3D7_0804400  *Pfmap* | GCTTCCTGTTGGGGTGTTT | AAAATTGTCATTTTGTTTAACACTT |
| PF3D7_1239700  *Pffmt* | GAGCAATTGAAAGGATGGAA | TTTTCCAAAACAACAAATAAAACA |
| PF3D7_1405700  *PfRING* | AAAATCCTCTTCGCACATTTTT | TTGATTATCACAAATGCTCATTCA |

**Supplementary File 1d: Oligos for generation and sequencing of allelic replacement constructs.**

| *Pfftsh1* primer | Forward primer | Reverse primer |
| --- | --- | --- |
| sgRNA | TAAGTATATAATATTTTCTGTTTTATTGCATGATGGTTTTAGAGCTAGAA | TTCTAGCTCTAAAACCATCATGCAATAAAACAGAAAATATTATATACTTA |
| Genome segment for allelic replacement | CCTAGGAAATGGGTGCTAGAATGCC | GCATGCAGCGACTAGTAAGAATATTTAT |
| sgRNA sequencing | AAAAAATTCTTGCTTGTTCAGA | CGGCCGCTATTTCATCTATTT |
| Shield mutations | CTTGAAGCACCACTACTTGTTTCAGATTTACCAAAAACAATTTCTTCAGC | GCTGAAGAAATTGTTTTTGGTAAATCTGAAACAAGTAGTGGTGCTTCAAG |
| G489C mutation | ATATATCACTTGAAGCACAACTACTTGTTTCAGATTTACCAAAAACAATTTCTTCAG | CTGAAGAAATTGTTTTTGGTAAATCTGAAACAAGTAGTTGTGCTTCAAGTGATATAT |
| Mutation sequencing | TGAAGCTGGTCATGCTATCG | TGATCTCTTCTCCGGATAAGG |
